# Observational anatomic study of ascending lumbar veins in human cadavers

**DOI:** 10.1590/1677-5449.202501552

**Published:** 2026-07-03

**Authors:** Felipe de Azevedo Correa Assumpção, Natália Santos dos Anjos, Carlos Adriano Silva dos Santos

**Affiliations:** 1 Universidade Estadual de Ciências da Saúde de Alagoas – UNCISAL, Maceió, AL, Brasil.

**Keywords:** anatomy, venous valves, collateral circulation, caval veins, iliac vein

## Abstract

**Background:**

The ascending lumbar veins (ALVs) and their continuations in the thorax as veins of the azygos and hemiazygos system constitute one of the main routes of collateral circulation in the caval system. However, there is some doubt about their efficiency, primarily with regard to the direction of flow and the presence or absence of valves in their lumina.

**Objectives:**

To conduct an anatomic study of the ALVs, noting the frequency, number, and types of valves present and the morphometry, tributaries, surrounding structures, drainage, and continuation of the ALVs.

**Methods:**

This is a descriptive anatomic study conducted with 15 human cadavers and a total of 30 dissections.

**Results:**

Six valves and four septa were observed. The ALVs most frequently drained into the common iliac vein (CIV) and in 40% of cases they did not continue longitudinally beyond the fourth lumbar vertebra. Muscular tributaries were the most frequent type. Mean ALV diameter was 2.75 mm on the right and 3.65 mm on the left. The ALVs were located lateral to the vertebral bodies and intervertebral discs; medial to the psoas major muscle and obturator nerve; posterior to the pelvic and abdominal lymph node chains and the CIV; and anterior to the transverse processes of the lumbar vertebrae.

**Conclusions:**

Considering the presence of valves and septa in the ALVs and the fact that they did not always extend beyond the fourth lumbar vertebra, it can be concluded that the ALVs cannot always be defined as routes of collateral circulation with ascending and descending flow supporting the caval system.

## INTRODUCTION

The ascending lumbar veins (ALVs) drain to the common iliac veins (CIVs). They are located between the psoas major muscle and the bases of the transverse processes of the lumbar vertebrae. Cranially, they join the subcostal veins, forming the azygos vein on the right and the hemiazygos vein on the left,^1^ which continue over the vertebral body of the twelfth thoracic vertebra and pass below the crura of the diaphragm to enter the thorax and drain into the superior vena cava. Additionally, the ALVs form anastomoses between the CIVs, the iliolumbar veins, and the lumbar veins.

In turn, the lumbar veins are four pairs of vessels that drain the abdominal walls and the spinal column, receiving veins from the vertebral venous plexuses and running horizontally towards their corresponding lumbar vertebral bodies. Before draining into the dorsal inferior vena cava, they are connected by the ALVs, which pass longitudinally of the vertebral bodies, forming the rich anastomotic network of the inferior vena cava.

In the same region, the inferior vena cava originates at the junction of the two CIVs and courses upwards, to the right of the aorta, draining into the right atrium. The inferior vena cava is a large-caliber vessel responsible for draining blood from the lower limbs, the abdominal walls and the digestive system.^2^

In conditions involving obstruction of the inferior vena cava, such as retroperitoneal tumors,^3^ deep venous thrombosis,^4^ and major bleeding requiring ligature during surgery^5^ or caused by abdominal traumas injuring the vessel, and also in individuals with congenital absence of the inferior vena cava,^2^ the system described above, formed by the ALVs, the azygos and hemizygos systems, and the vertebral venous plexuses, serves as one of the most important direct or indirect routes of collateral communication between lower limbs, abdominal walls, and pelvis, enabling drainage to the superior vena cava. However, there are few publications in the literature describing presence or absence of valves in these veins and descriptions of their tributaries are ambiguous. Therefore, the present study is intended to meet the need for more detailed anatomic descriptions of the ALVs as a valveless system that may be capable of bidirectional flow and, therefore, serve as a collateral communication between the inferior and superior caval systems, making it possible to depend on this anastomotic network during surgical procedures and in clinical practice.

The objective of this study is to conduct an anatomical description of the ALVs in human cadavers, covering the frequency, number, and types of valves and the characteristics of their tributaries, morphometry, surrounding structures, drainage, and continuation.

## METHODS

The study was approved by the Research Ethics Committee at the Universidade Estadual de Ciências da Saúde de Alagoas (UNCISAL), under CAAE submission number 80872024.0.0000.5011 and opinion number 7.007.774.

This study was supported by the Alagoas State Research Funding Agency (Fundação de Amparo à Pesquisa do Estado de Alagoas - FAPEAL) via its undergraduate bursary program (Programa Institucional de Bolsas para Iniciação Científica - PIBIC), funding round PIBIC 202- /2025. Study duration was 1 year (12 months).

This is a macroscopic, descriptive, anatomic study of human cadavers preserved in formaldehyde solution. It was conducted in the anatomy laboratory at UNCISAL. The sample was non-systematic, by convenience, in that the sample was assembled according to the availability of cadavers provided for students at the anatomy laboratory. The sample comprised 15 cadavers with intact abdominal-pelvic and thoracic cavities on both sides, totaling 30 dissection samples.

In view of the absence of descriptions or evidence on the presence of valves in the ALVs that could be used as a basis for calculating sample size, the study used an article by Lu et al.,^6^ which employed 15 cadavers (totaling 30 samples) with the objective of conducting an anatomic study of ALVs. Based on the proportions actually observed in the present study, an *a posteriori* calculation using the procedure for estimating proportions described by Miot^7^ and adopting a 95% confidence level (Z=1.96) estimated the absolute sample error as 17%.

**Figure 1:**
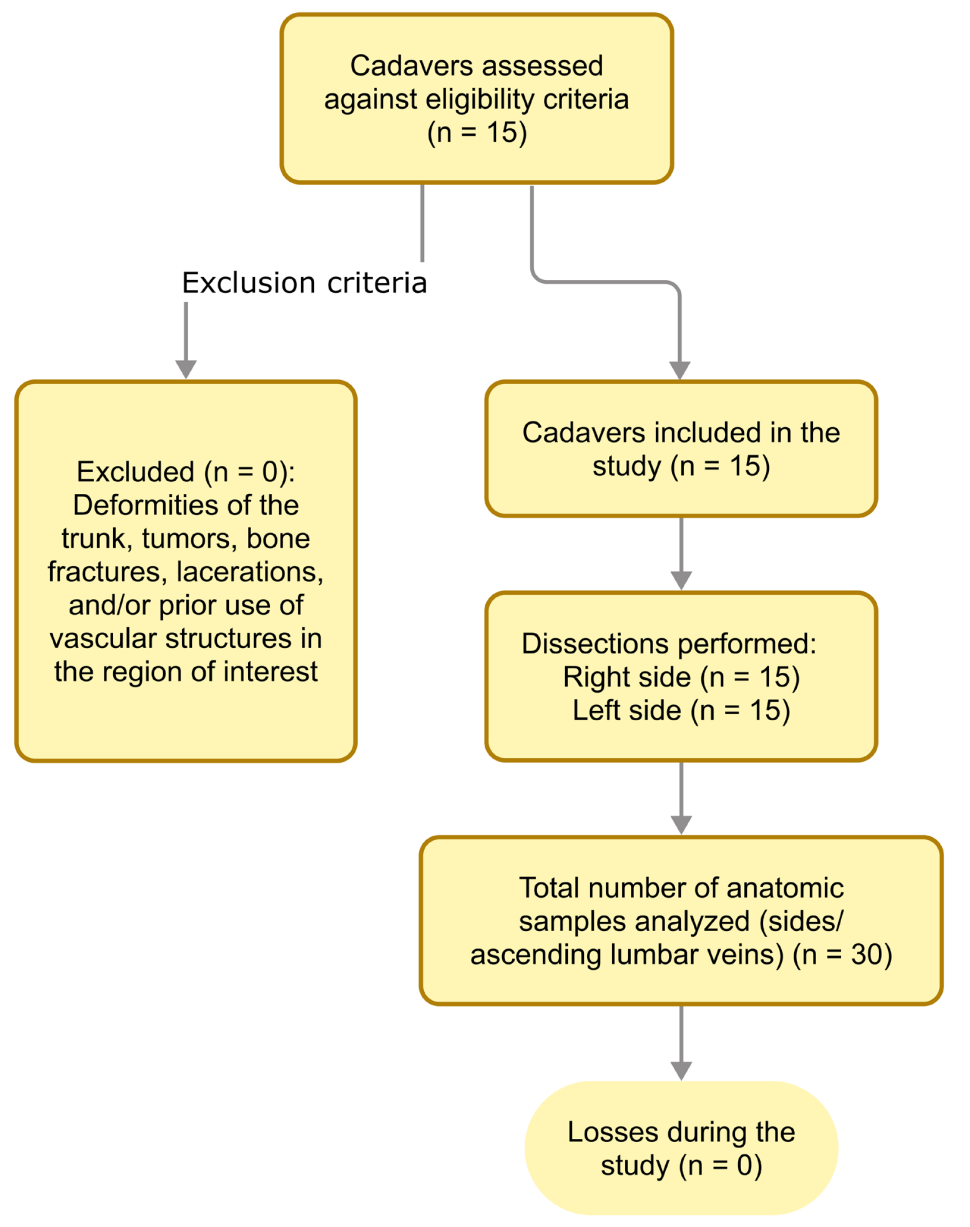
Flowchart of sample inclusions, exclusions and losses.

The sample comprised male cadavers, aged from 18 to 65 years, duly preserved in formaldehyde and with both sides of the abdominal-pelvic and thoracic cavities still attached to the axial skeleton, in which the ALVs were present and intact. The exclusion criteria were cadavers with deformities involving the trunk, such as tumors, bone fractures, laceration, or prior use of the vascular and nerve structures related to the segment being studied, since such deformities distort the anatomic relationships of the vessel under analysis and could compromise the results of the research. However, it did not prove necessary to exclude any of the cadavers (Figure 1).

The dissection technique consisted of positioning the cadaver in the supine position, with the abdominal-pelvic and thoracic cavities exposed in advance. The region of interest was dissected with great care, exposing the structures to be analyzed, in conjunction with a mesoscopic inspection of the abdominal and thoracic compartments.

After the area had been exposed, the ALVs were analyzed along their entire length to describe their drainage and continuation. Additionally, their surrounding structures were also examined, in order to analyze their physical relationships.

For the analysis of ALV valves, a longitudinal incision was made in each ALV, exposing the interior, to enable the frequency, number, and types of valves present to be observed. The data obtained were recorded in tables on the study instrument.

All morphometric data were obtained using a digital pachymeter (Mitutoyo Digimatic Caliper; Mitutoyo America Corporation, Aurora, United States). Data were expressed in percentages, and morphometric data were expressed as means, medians, and standard deviations. Data were compiled and analyzed in spreadsheets using Google Sheets.

The EQUATOR group’s STROBE (Strengthening the Reporting of Observational Studies in Epidemiology) checklist for observational studies and AQUA (Anatomical Quality Assurance) checklist for anatomic studies were used to guide the study.

According to Law No. 8.501, of November 30, 1992, cadavers that are not claimed from the authorities within 30 days can be used for educational and research purposes. There was therefore no need for free and informed consent, since the laboratories involved took responsibility for the cadavers studied.

## RESULTS

**Figure 2:**
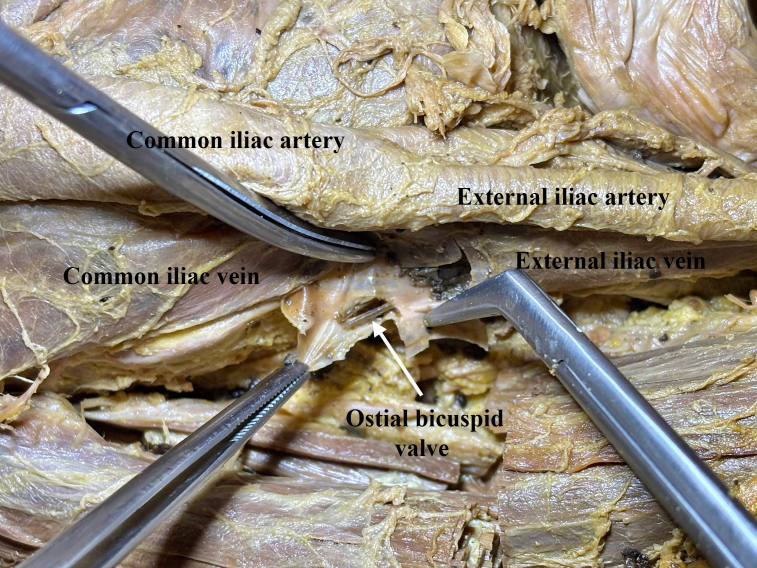
Ostial bicuspid valve present in the right ascending lumbar vein.

**Figure 3:**
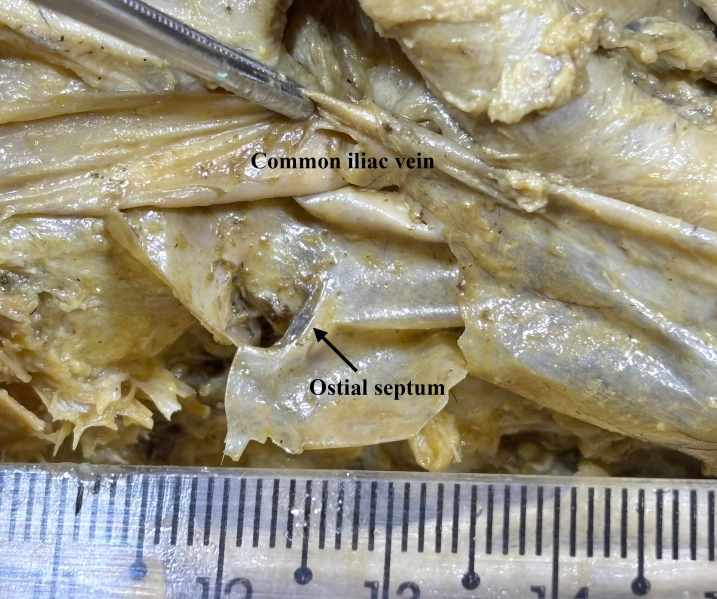
Ostial septum present in the ascending lumbar vein.

Nine of the 15 cadavers (60%) had valves or septa in the ALV on at least one side. One (6.6%) cadaver had valves on both sides, bicuspid on the right (Figure 2) and unicuspid on the left. The remaining eight (53.3%) cadavers had unilateral valves or septa (Figure 3).

**Figure 4:**
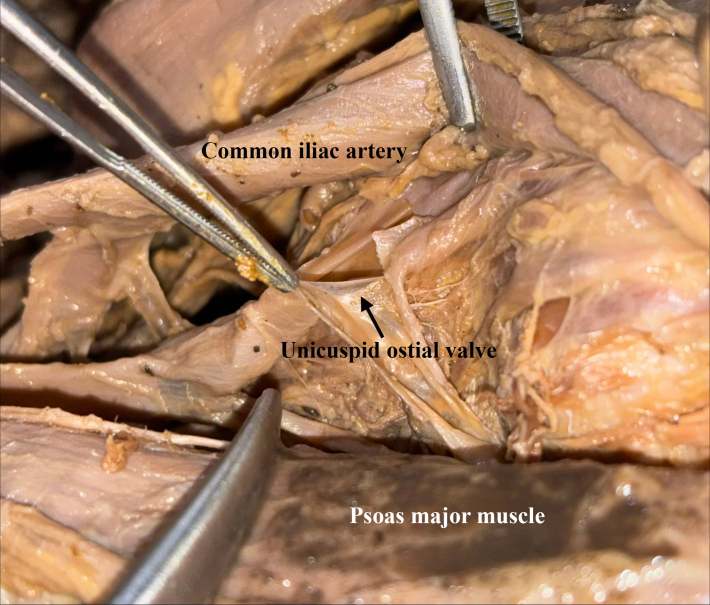
Ostial unicuspid valve present in the ascending lumbar vein.

Considering the 30 sides analyzed, 10 had valves or septa. Four of these (40%) had unicuspid valves (Figure 4), two (20%) had bicuspid valves, and four (40%) had ostial septa. All of the valves were ostial, i.e., they were located at the ALV drainage ostium (Table 1).

**Table 1:** Frequency of valves and septa in the ascending lumbar vein and their classifications in terms of type of cuspid and distribution by side.

	**Unicuspid valve**	**Bicuspid valve**	**Septum**
Right side	1	1	3
Left side	3	1	1

Muscular veins were the most common type of ALV tributary, followed by the fourth lumbar vein (Table 2).

**Table 2:** Tributaries of ascending lumbar vein per side.

	**Fourth lumbar vein**	**Fifth lumbar vein**	**Iliolumbar vein**	**Muscular veins**	**Anastomotic tributaries**
Right side	9	8	4	12	5
Left side	13	10	7	12	3

There was great variability in ALV drainage patterns (Table 3). On the left side, drainage into the CIV was most frequent, in 11 of 15 (73.3%) cases. Drainage into the CIV was also most frequent on the right side, in seven (46.6%) cases, followed by drainage into a common trunk formed with the iliolumbar vein, in five (33.3%) cases. In 12 out of 30 sides (40%), the ALV did not extend beyond the fourth lumbar vertebra (L4) (Table 4).

**Table 3:** Drainage of ascending lumbar veins by side.

	**Common iliac**	**External iliac**	**Internal iliac**	**Common trunk formed with iliolumbar vein**
Right side	7	2	1	5
Left side	11	2	0	2

**Table 4:** Continuation of ascending lumbar vein beyond the fourth lumbar vertebra (L4) by side.

	**Continues after L4**	**Does not continue after L4**
Right side	7	8
Left side	11	4

Morphometry results showed that the diameter of the left ALVs was greater than the diameter of the right ALVs (Table 5).

**Table 5:** Morphometry of the ascending lumbar vein, illustrated by statistical analysis of diameter at drainage.

**Sample**	**Right side**	**Left side**
Mean	2.75 mm	3.65 mm
Median	2.7 mm	4.14 mm
Standard deviation	1.17	1.24

With relation to the surrounding structures, in the 15 cadavers studied, the vertebral bodies and intervertebral discs were medial to the right ALVs in all cases. The psoas major muscle was identified lateral to the right ALV in 15 cases (100%) and the obturator nerve was lateral in 11 cases (73.3%). The psoas major muscle and the pelvic and abdominal lymph node chains were observed anterior to the right ALV in 15 (100%) cases and the CIV was anterior in 14 (93.3%) cases. The transverse processes of the lumbar vertebrae were posterior to the right ALV in all cases.

On the left side, the vertebral bodies and intervertebral discs were also medial to the ALV in all cadavers. The psoas major muscle was lateral in all cases, the femoral nerve was lateral in seven (46.6%), and the obturator nerve was lateral to the left ALV in 13 (86.6%) cases. The pelvic and abdominal lymph node chains were anterior in all cases and the psoas major muscle was anterior to the left ALV in 13 (86.6%) cases. Finally, the transverse processes of the lumbar vertebrae were observed posterior to the left ALV in 14 (93.3%) cases, while the nerve roots of the L4 lumbosacral plexus in eight (53.3%) cases and the L5 lumbosacral plexus nerve roots in nine (60%) cases were observed posterior to the left ALV.

## DISCUSSION

Some studies, such as one by Araújo et al.,^5^ have described the ALV as a possible route of collateral communication for the caval system. However, there is a gap in the literature with regard to the presence or absence of valves in the ALV, which could directly compromise this collateral communication.

In the present study, valves or septa capable of affecting blood flow were observed in nine cadavers. In four of these cadavers, valves were observed that would obstruct flow in the ascendent lumbar direction, directing it to the CIV or the common trunk formed with the iliolumbar vein. In one of these cases, a unicuspid valve was identified that would impede flow from the ALV to the CIV, redirecting it to the ALV.

Another relevant finding was that the ALV extended beyond L4 in just 18 of the 30 (60%) sides examined and the ALV only continued beyond this level with no valves or septa that would obstruct blood flow in just eight of these (44.4%). As such, just 10/30 (33.3%) of the ALVs analyzed did not exhibit any impediment to collateral flow up to the segment studied. While there are reports, such as that by Araújo et al.,^5^ describing total venous return via the ALV, extending as far as the azygos system and draining into the superior vena cava, the results of the present study indicate that depending solely and exclusively on this collateral system, without verifying its patency in advance, could lead to compromised circulation. However, in order to define collateral circulation, in addition to anatomy, it is necessary to study local pathophysiology, since diseases such as venous hypertension and chronic venous insufficiency are associated with valve incompetence,^8^ increased vein caliber and expansion of collateral networks. It is therefore important to conduct studies to investigate these conditions, correlating them with anatomic findings.

The literature is controversial with regard to the caudal segment of the ALV, questioning whether it constitutes its origin or its point of drainage, and also which structures it connects to. Classical anatomic texts describe this part of the ALV as connecting to the CIV. Lolis et al.,^1^ in turn, report that it is possible to find the ALV draining to the CIV, the external iliac vein (EIV), the internal iliac vein (IIV), or to a common trunk formed with the iliolumbar vein.

Over the course of the present study, it was determined that, according to the vein’s drainage pattern and the direction of flow imposed by the valves observed, the ALV is formed by its tributaries, which are therefore responsible for its origin. However, in order to be able to define the origin of these veins, embryological studies are needed to investigate this aspect in depth.

Moreover, it was found that the ALV most frequently drains to the CIV, although patterns of drainage to the EIV and IIV and to a common trunk formed with the iliolumbar vein were also observed. These results corroborate Lolis et al.,^1^ with respect to the different possibilities of ALV drainage in addition to into the CIV.

The principal limitation of this study was its sample size, which is because of limited availability of cadavers. Therefore, it is suggested that future studies should analyze larger samples to be able to assess the distribution of variables in different ethnic groups, age groups, and sexes.

Based on the data obtained in the present study, it can be concluded that ALVs may contain valves or septa and sometimes do not extend beyond L4, which could compromise collateral circulation if they are used as a route of collateral communication with the caval system. These veins are formed by their tributaries and most frequently drain to the CIV. They have a larger diameter on the left side and are related, medially, with the vertebral bodies and intervertebral discs; laterally with the psoas major muscle and obturator nerve; anteriorly, with the psoas major muscle, the pelvic and abdominal lymph node chains and the CIV; and, posteriorly, with the transverse processes of the lumbar vertebrae.

## Data Availability

Os dados que sustentam este estudo estão disponíveis mediante solicitação ao autor correspondente, FACA.

## References

[1] Lolis E, Panagouli E, Venieratos D (2011). Study of the ascending lumbar and iliolumbar veins: surgical anatomy, clinical implications and review of the literature. Ann Anat.

[2] Alves EC, Ferro GBR, França LKL, Jacó MB, Pitta GBB (2010). Ausência de veia cava inferior: relato de caso. J Vasc Bras.

[3] Castro IF, Tustumi F, Yamashita ET (2023). Ressecção cirúrgica de leiomiossarcoma de veia cava inferior retro-hepática sem reconstrução vascular: relato de caso. J Vasc Bras.

[4] Onzi RR, Costa LF, Angnes RF (2007). Malformação de veia cava inferior e trombose venosa profunda: fator de risco de trombose venosa em jovens. J Vasc Bras.

[5] Araújo J, Mendes D, Vilar VS, Franco J (1979). Sobre um caso de ligadura e ressecção da veia cava inferior, em leiomiossarcoma retro-peritonial. Acta Oncol Bras..

[6] Lu S, Xu Y, Ding Z, Wang Y, Shi J, Zhong S (2008). Clinical anatomic study of the lower lumbar anterolateral vein: with respect to retroperitoneal endoscopic surgery. Chin J Traumatol.

[7] Miot HA (2011). Tamanho da amostra em estudos clínicos e experimentais. J Vasc Bras.

[8] França LHG, Tavares V (2003). Insuficiência venosa crônica: uma atualização. J Vasc Bras.

