# Supplementary material for: Observational anatomic study of ascending lumbar veins in human cadavers
Source: J Vasc Bras. 2026 Jul 3;25:e20250155. doi: 10.1590/1677-5449.202501552 (PMC13340972; doi:10.1590/1677-5449.202501552)
Supplement: Translation

# Translation: Estudo anatômico observacional de veias lombares ascendentes em cadáveres humanos

**DOI:** 10.1590/1677-5449.202501551

**Authors:** Felipe de Azevedo Correa Assumpção, Natália Santos dos Anjos, Carlos Adriano Silva dos Santos

**Affiliations:** 1 Universidade Estadual de Ciências da Saúde de Alagoas – UNCISAL, Maceió, AL, Brasil.

**Keywords:** anatomia, válvulas venosas, circulação colateral, veias cavas, veia ilíaca

## Abstract

**Contexto:**

As veias lombares ascendentes (VLAs) e sua continuação no tórax como veias do sistema ázigo e hemiázigo consistem em uma das principais vias de circulação colateral do sistema caval. Porém, existem dúvidas quanto à sua eficiência, sobretudo quanto ao direcionamento do fluxo decorrente da presença ou não de válvulas em seu lúmen.

**Objetivos:**

Estudar anatomicamente as VLAs quanto à frequência, ao número e aos tipos de válvulas, bem como sua morfometria, tributárias, sintopia, desembocadura e continuidade.

**Métodos:**

Trata-se de estudo anatômico descritivo realizado em cadáveres humanos, no qual foram incluídos 15 cadáveres, totalizando 30 dissecções.

**Resultados:**

Foram observados seis válvulas e quatro septos. A desembocadura da VLA ocorreu com maior frequência na veia ilíaca comum (VIC), e não houve continuidade longitudinal após a quarta vértebra lombar em 40% dos casos. As tributárias musculares foram as mais frequentes. A VLA apresentou diâmetro médio de 2,75 mm no lado direito e de 3,65 mm no lado esquerdo. Encontrava-se lateralmente aos corpos vertebrais e discos intervertebrais; medialmente ao músculo psoas maior e nervo obturador; posteriormente à cadeia linfonodal parietal abdominopélvica e à VIC; e anteriormente aos processos transversos das vértebras lombares.

**Conclusões:**

Considerando a presença de válvulas e septos nas VLAs e o fato de que nem sempre houve continuidade além da quarta vértebra lombar, conclui-se que as VLAs não podem sempre ser definidas como vias de circulação colateral com fluxo ascendente e descendente em apoio ao sistema caval.

## INTRODUÇÃO

As veias lombares ascendentes (VLAs) desembocam nas veias ilíacas comuns (VICs). Localizam-se entre o músculo psoas maior e as raízes dos processos transversos das vértebras lombares. Cranialmente, ao se unirem à veia subcostal, formam a veia ázigo à direita e a veia hemiázigo à esquerda^1^, as quais se continuam sobre o corpo da 12ª vértebra torácica e passam abaixo dos pilares do diafragma para adentrar o tórax e desembocar na veia cava superior. Ademais, as VLAs estabelecem anastomoses entre as VICs, as veias iliolombares e as veias lombares.

Em relação às veias lombares, estas se apresentam em quatro pares que drenam as paredes abdominais e a coluna vertebral, da qual recebem veias dos plexos vertebrais, e seguem horizontalmente em direção aos corpos das vértebras lombares correspondentes. Antes de desembocarem na parte dorsal da veia cava inferior, são conectadas pela VLA, que passa longitudinalmente aos corpos vertebrais, formando essa rica rede anastomótica da veia cava inferior.

Nesse contexto, a veia cava inferior se origina da junção das VICs e segue de maneira ascendente, à direita da artéria aorta, para desembocar no átrio direito. Trata-se de um vaso de grande calibre, responsável por drenar o sangue proveniente dos membros inferiores, das paredes abdominais e do sistema digestório^2^.

Em casos de obstrução da veia cava inferior, como em neoplasias retroperitoneais^3^, trombose venosa profunda^4^, necessidade de ligadura por grande sangramento cirúrgico^5^ ou traumas abdominais com lesão desse vaso, bem como em casos de ausência congênita da veia cava inferior^2^, o sistema supracitado, que envolve a VLA na formação do sistema ázigo e hemiázigo, além dos plexos venosos vertebrais, apresenta-se como uma das principais vias de colateralidade direta ou indireta entre membros inferiores, parede abdominal e pelve, permitindo o escoamento para a veia cava superior. Entretanto, ainda são escassas as descrições na literatura acerca da presença ou não de válvulas nessas veias, além de haver ambiguidade na descrição de suas tributárias. Portanto, o presente estudo busca satisfazer a necessidade de descrições anatômicas mais detalhadas das VLAs como um sistema avalvulado, que possa apresentar fluxo bidirecional e, assim, servir como via colateral entre os sistemas caval inferior e superior, determinando a possibilidade de dependência dessa rede anastomótica tanto em procedimentos cirúrgicos como na clínica médica.

O objetivo deste estudo é descrever anatomicamente as VLAs, em cadáveres humanos, quanto à frequência, ao número e ao tipo de válvulas, bem como caracterizar suas tributárias, morfometria, sintopia, desembocadura e continuidade.

## MÉTODOS

O estudo foi aprovado pelo Comitê de Ética em Pesquisa da Universidade Estadual de Ciências da Saúde de Alagoas (UNCISAL), sob o número CAAE 80872024.0.0000.5011 e parecer 7.007.774.

A pesquisa contou com o fomento da Fundação de Amparo à Pesquisa do Estado de Alagoas (FAPEAL), por meio do Programa Institucional de Bolsas para Iniciação Científica (PIBIC), com início conforme o calendário PIBIC 2024/2025. O estudo teve a duração de 1 ano (12 meses).

Trata-se de um estudo anatômico descritivo macroscópico utilizando cadáveres humanos conservados em solução de formaldeído. O estudo foi realizado no laboratório de anatomia da UNCISAL. Utilizou-se a técnica de amostragem não sistemática por conveniência, sendo a amostra definida de acordo com a disponibilidade de cadáveres destinados aos alunos do laboratório de anatomia. A amostra foi composta por 15 cadáveres com ambos os dimídios das cavidades abdominopélvica e torácica, totalizando 30 amostras.

Devido à ausência de descrições ou evidências da presença de válvulas na VLA que fundamentassem o cálculo prévio do tamanho amostral, este estudo tomou como referência o artigo de Lu et al.^6^, que utilizou 15 cadáveres (totalizando 30 amostras) com o objetivo de estudar anatomicamente as VLAs. Com base nas proporções observadas no presente estudo, estimou-se, *a posteriori*, por meio da expressão para estimativas de proporções descritas por Miot^7^, adotando-se um nível de confiança de 95% (Z=1,96), que a amostra apresenta erro absoluto de 17%.

Foram incluídos cadáveres do sexo masculino, com idades entre 18 e 65 anos, devidamente formolizados e com ambos os dimídios das cavidades abdominopélvica e torácica articulados ao esqueleto axial, nos quais as VLAs estivessem presentes e íntegras. Os critérios de exclusão foram cadáveres com deformidades no tronco, como tumores, fratura óssea, laceração ou utilização prévia de estruturas vasculares e nervosas relacionadas ao segmento em estudo, pois tais deformidades distorcem as relações anatômicas do vaso analisando, podendo comprometer os resultados da pesquisa. No entanto, não houve a necessidade de exclusão de nenhum cadáver (Figura 1).

**Figura 1:** Fluxograma de inclusões, exclusões e perdas da amostra.

A técnica de dissecação consistiu em posicionar o cadáver em decúbito dorsal, com as cavidades abdominopélvica e torácica previamente expostas. Realizou-se dissecção minuciosa da região de interesse, a fim de expor as estruturas a serem avaliadas, em conjunto com uma inspeção mesoscópica dos compartimentos abdominal e torácico.

Após exposição da região, as VLAs foram analisadas ao longo de seu trajeto para descrever sua desembocadura e continuidade. Além disso, foram avaliadas suas estruturas circunvizinhas, a fim de analisar a sintopia das VLAs.

Para a análise das válvulas, realizou-se uma incisão longitudinal nas VLAs, expondo seu interior para avaliação da frequência, do número e dos tipos de válvulas. Os dados obtidos foram registrados em tabelas no instrumento de pesquisa.

Todas as medidas morfométricas foram obtidas por intermédio de um paquímetro digital (Mitutoyo Digimatic Caliper; Mitutoyo America Corporation, Aurora, EUA). Os dados foram representados em porcentagem, e as medidas morfométricas foram expressas em média, mediana e desvio-padrão. Os dados foram compilados e analisados em planilhas produzidas no programa Google Sheets.

Foram utilizados o checklist STROBE (*Strengthening the Reporting of Observational Studies in Epidemiology*) para estudos observacionais e o checklist AQUA (*Anatomical Quality Assurance*) para estudos anatômicos, da rede EQUATOR, para guiar e orientar a elaboração do presente estudo.

Segundo a Lei Ordinária n.º 8.501, de 30 de novembro de 1992, cadáveres não reclamados junto às autoridades no prazo de 30 dias podem ser destinados a fins de ensino e pesquisa. Assim, não houve necessidade de solicitação de termo livre e esclarecido, haja vista que os laboratórios envolvidos assumiram a responsabilidade pelos cadáveres estudados.

## RESULTADOS

Dos 15 cadáveres, nove (60%) apresentaram, em pelo menos um dos lados, válvula ou septação. Um (6,6%) cadáver apresentou válvula em ambos os lados, sendo bicúspide (Figura 2) à direita e unicúspide à esquerda. Os oito (53,3%) cadáveres restantes apresentaram válvulas ou septos (Figura 3) unilateralmente.

**Figura 2:** Presença de válvula bicúspide ostial na veia lombar ascendente direita.

**Figura 3:** Presença de septo ostial na veia lombar ascendente.

Considerando os 30 dimídios analisados, 10 apresentaram válvulas ou septos. Desses, quatro (40%) apresentaram válvula unicúspide (Figura 4), dois (20%) apresentaram válvula bicúspide e quatro (40%), septos ostiais. Todas as válvulas identificadas eram do tipo ostial, ou seja, localizavam-se no óstio de desembocadura da VLA (Tabela 1).

**Figura 4:** Presença de válvula unicúspide na veia lombar ascendente.

**Tabela 1:** Frequência de válvulas e septos na veia lombar ascendente e sua classificação quanto ao tipo de cúspide e distribuição por lado.

	**Válvula unicúspide**	**Válvula bicúspide**	**Septo**
Lado direito	1	1	3
Lado esquerdo	3	1	1

Nas VLAs, as tributárias musculares foram as mais frequentes, seguidas da quarta veia lombar (Tabela 2).

**Tabela 2:** Tributárias da veia lombar ascendente por lado.

	**Quarta veia lombar**	**Quinta veia lombar**	**Veia iliolombar**	**Musculares**	**Tributárias anastomóticas**
Lado direito	9	8	4	12	5
Lado esquerdo	13	10	7	12	3

Observou-se grande variabilidade quanto à desembocadura das VLAs (Tabela 3). No lado esquerdo, a desembocadura ocorreu mais frequentemente na VIC, em 11 de 15 (73,3%) casos. No lado direito, a desembocadura também foi mais frequente na VIC, em sete (46,6%) casos, seguida por desembocadura em tronco comum com a veia iliolombar, em cinco (33,3%) casos. A VLA não se continuou depois da quarta vértebra lombar (L4) em 12 dos 30 (40%) lados (Tabela 4).

**Tabela 3:** Desembocadura da veia lombar ascendente por lado.

	**Ilíaca comum**	**Ilíaca externa**	**Ilíaca interna**	**Tronco comum com veia iliolombar**
Lado direito	7	2	1	5
Lado esquerdo	11	2	0	2

**Tabela 4:** Continuidade da veia lombar ascendente após a quarta vértebra lombar (L4) por lado.

	**Continua depois de L4**	**Não continua depois de L4**
Lado direito	7	8
Lado esquerdo	11	4

Quanto à morfometria, observou-se que o diâmetro das VLAs foi maior no lado esquerdo (Tabela 5).

**Tabela 5:** Morfometria da veia lombar ascendente, representada pela análise estatística de seu diâmetro na desembocadura.

**Amostra**	**Lado direito**	**Lado esquerdo**
Média	2,75 mm	3,65 mm
Mediana	2,7 mm	4,14 mm
Desvio-padrão	1,17	1,24

Em relação à variável sintopia, nos 15 cadáveres estudados, observou-se que, no lado direito, os corpos vertebrais e discos intervertebrais estavam mediais às VLAs em todos os casos. Lateralmente, o músculo psoas maior foi identificado em 15 (100%) casos e o nervo obturador, em 11 (73,3%). Anteriormente, o músculo psoas maior e a cadeia linfonodal parietal abdominopélvica foram observados em 15 (100%) casos e a VIC, em 14 (93,3%). Posteriormente, os processos transversos das vértebras lombares estavam presentes em todos os casos.

No lado esquerdo, os corpos vertebrais e os discos intervertebrais também se encontravam mediais às VLAs em todos os cadáveres. Lateralmente, o músculo psoas maior foi identificado em todos os casos, o nervo femoral, em sete (46,6%) e o nervo obturador, em 13 (86,6%). Anteriormente, observaram-se o músculo psoas maior em 13 (86,6%) e a cadeia linfonodal parietal abdominopélvica em todos os casos. Posteriormente, identificaram-se os processos transversos das vértebras lombares em 14 (93,3%), além das raízes nervosas do plexo lombossacral L4 em oito (53,3%) e L5 em nove (60%).

## DISCUSSÃO

Alguns estudos, como o de Araújo et al.^5^, descrevem a VLA como uma possível via de colateralidade para o sistema caval. No entanto, a literatura apresenta-se bastante omissa quanto à presença ou não de válvulas na VLA, o que pode prejudicar diretamente essa via de colateralidade.

No presente estudo, observaram-se, em nove cadáveres, válvulas ou septos capazes de influenciar o fluxo sanguíneo. Em quatro desses cadáveres, observaram-se válvulas que dificultavam o fluxo no sentido lombar ascendente, direcionando-o à VIC ou ao tronco comum com a veia iliolombar. Em um desses casos, identificou-se uma válvula unicúspide que impedia o fluxo da VLA para a VIC, redirecionando-o à VLA.

Outro achado relevante foi que apenas 18 dos 30 (60%) lados apresentaram continuidade da VLA após a L4 e, desses 18, apenas oito (44,4%) continuavam após esse nível sem apresentar válvulas ou septos que impedissem o fluxo sanguíneo. Sendo assim, apenas 10/30 (33,3%) das VLAs analisadas não apresentaram nenhum impedimento para a colateralidade do fluxo até o segmento estudado. Embora existam relatos, como o de Araújo et al.^5^, descrevendo retorno venoso pleno por meio da VLA com continuidade no sistema ázigo e desembocadura na veia cava superior, os resultados deste estudo indicam que a dependência única e exclusiva desse sistema colateral, sem que se antes verifique sua perviedade, pode resultar em prejuízos circulatórios. Entretanto, para que se possa definir a circulação colateral, além da anatomia, deve-se realizar um estudo sobre a patofisiologia local, uma vez que doenças como hipertensão venosa e insuficiência venosa crônica estão associadas a incompetência valvar^8^, aumento do calibre de veias e expansão de redes colaterais. Nesse sentido, é preciso que estudos futuros abordem tais moléstias, correlacionando-as com achados anatômicos.

A literatura é controversa quanto à porção caudal da VLA, questionando se esta corresponde à sua origem ou à sua desembocadura, bem como quanto à estrutura à qual se conecta. As literaturas anatômicas clássicas descrevem essa porção da VLA conectada à VIC. Lolis et al.^1^, por sua vez, descrevem que há possibilidade de desembocadura da VLA na VIC, na veia ilíaca externa (VIE), na veia ilíaca interna (VII) ou em tronco comum com a veia iliolombar.

Ao longo deste estudo, identificou-se que, pelo padrão de drenagem dessa veia e pelo direcionamento do fluxo imposto pelas válvulas identificadas, a VLA é formada pelas suas tributárias, as quais seriam, portanto, responsáveis por sua origem. Entretanto, para que se possa definir a origem dessas veias, são necessários estudos embriológicos que investiguem mais profundamente esse aspecto.

Além disso, verificou-se que a VLA desemboca com maior frequência na VIC, embora também tenham sido observadas desembocaduras na VIE, na VII e em tronco comum com a veia iliolombar. Esses resultados corroboram com Lolis et al.^1^ quanto às diferentes possibilidades de desembocadura da VLA além da VIC.

A principal limitação deste estudo foi o tamanho da amostra, decorrente do acesso limitado a cadáveres. Dessa forma, sugere-se que pesquisas futuras ampliem o número da amostra para poder avaliar a distribuição das variáveis em diferentes grupos étnicos, faixas etárias e sexos.

Com base nos dados obtidos no presente estudo, conclui-se que as VLAs podem apresentar válvulas ou septos e que, por vezes, não se continuam após a L4, o que pode comprometer a circulação colateral caso sejam utilizadas como via de colateralidade para o sistema caval. São veias formadas a partir de suas tributárias e desembocam com maior frequência na VIC. Apresentam maior diâmetro no lado esquerdo e relacionam-se, medialmente, com os corpos vertebrais e discos intervertebrais; lateralmente, com o músculo psoas maior e o nervo obturador; anteriormente, com o músculo psoas maior, a cadeia linfonodal parietal abdominopélvica e a VIC; e, posteriormente, com os processos transversos das vértebras lombares.

## Data Availability

The data supporting this study are available on request from the corresponding author, FACA.
